# Detection of Postcoronary Stent Complication: Utility of 64-Slice Multidetector CT 

**DOI:** 10.1155/2012/214760

**Published:** 2012-07-01

**Authors:** Alpa Bharati, Suleman Merchant, Tilak Suvarna, Neha Parashar

**Affiliations:** ^1^Derpartment of Radiology, LTM General Hospital, Mumbai 400022, India; ^2^Derpartment of Radiology, Asian Heart Institute, Mumbai 400051, India

## Abstract

Coronary stent fracture is a known complication of coronary arterial stent placements. Multiple long-term risks are also associated with drug eluting stents. 64-slice multidetector CT (MDCT) coronary angiography has been shown to detect poststent complications such as instent stenosis, thrombosis, stent migration and stent fractures. We report a case of stent fracture in a patient who underwent RCA stenting with associated RCA perforation and almost complete thrombosis of the RCA and peristent fibrinoid collection. This is a rare case of stent fracture with perforation of the RCA. The paper highlights the role of 64-row multidetector computed tomography (MDCT) in evaluation of such poststent placement complications.

## 1. Introduction

Stent fracture (SF) has been suggested as one of the leading risk factors of thrombosis and instent restenosis (ISR) in patients who have intracoronary drug-eluting stent (DES) implants [1]; however, it may be easily missed due to its nonspecific presentation [2]. Recent reports suggest that the prevalence of fracture ranges between 1.9% and 2.6% [3–5]. 64-detector-row MDCT coronary angiography has proved to be highly accurate in the detection of coronary artery stenosis [6, 7], due to its high spatial and temporal resolution [1]. This feature can be utilized extremely well in evaluating poststenting complications too. Our case demonstrates two fractures in a single mid RCA, stent, implanted approximately two years prior to CT coronary angiography. The stent revealed a loss of normal alignment, perforation of the mid RCA and stent migration outside the RCA. The in-stent RCA was consequently completely occluded with minimal flow in a short segment of post-stent RCA and in its acute marginal branch vessel. Collateral circulation through posterior descending artery (PDA) was also demonstrated on CT coronary angiography. It is hence a rare case of stable RCA perforation following stent placement. This case also highlights the role of CT coronary angiography (CTCA) in detecting stent fracture and associated coronary occlusion and perforation.

## 2. Case Reports

**Figure 1:**
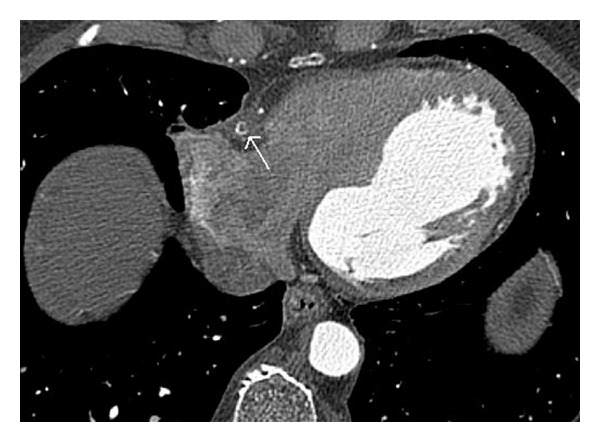
Nonenhancing fluid collection (arrow) around the RCA stent.

**Figure 2:**
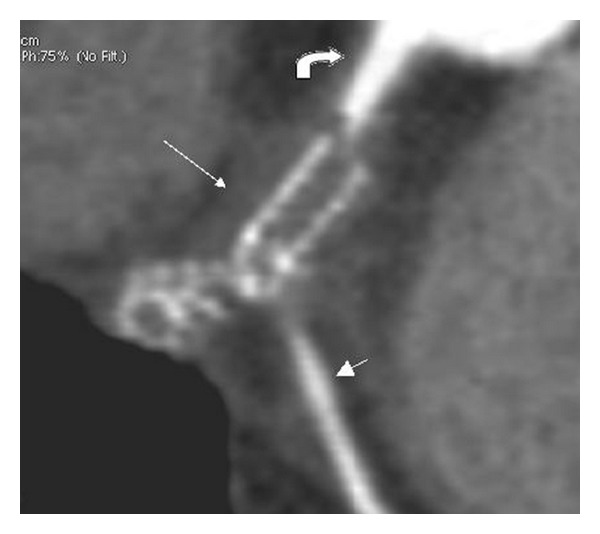
Hypodense fluid collection (long arrow) around the fractured stent, with perforation of RCA (curved arrow). Hypodensity suggestive of instent thrombosis is also seen. Also note good opacification of the acute marginal branch of the RCA (short arrow).

**Figure 3:**
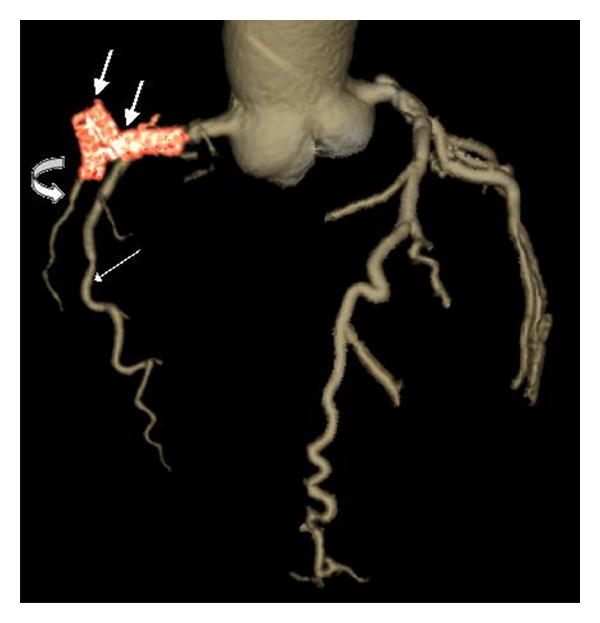
Volume-rendered image reveals RCA stent fracture at two levels (short thick arrows) with RCA perforation. Note opacified acute marginal branch of the RCA (long white arrow) and a faintly opacified poststent RCA (curved arrow).

**Figure 4:**
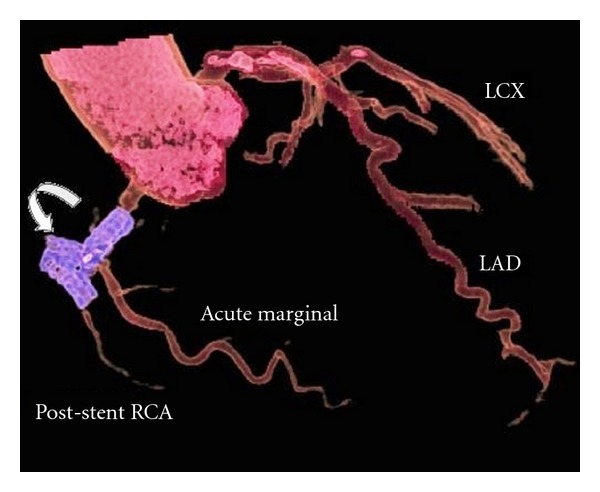
Volume-rendered image showing fractured RCA stent perforating the RCA (curved arrow), followed by faint poststent opacification of the RCA. Good opacification of LAD, LCX, and the acute marginal branch of the RCA is noted.

A 65-year-old male patient presented with a tuberculous knee joint effusion and occasional chest pain. He also had a drug eluting stent inserted in the mid RCA, two years back. However, the patient had recurrent angina within a month of stent placement. A catheter angiography done at that time revealed total occlusion of the RCA stent, with grade III collaterals. He was now referred to our department with a request for CT chest to exclude pulmonary tuberculosis. The CT chest revealed no signs of pulmonary tuberculosis but revealed an irregular localised pericardial fluid collection along the RCA stent (Figure 1). A dedicated CT coronary angiography was thus performed. This revealed a 1.5 × 2.0 cm sized, nonenhancing, fluid collection around the RCA, at the level of the stent. The stent itself was bent at two sites, with a complete fracture in its mid part (Figures 2 and 3). Additionally, the stent had perforated the RCA and migrated across the wall of the RCA; to partially lie in the pericardial sac, and was surrounded by a fluid collection/thrombus (Figure 2). The stent was completely thrombosed and the distal post-stent RCA showed faint opacification beyond the fractured site (Figures 3 and 4). Good opacification of the acute marginal branch was noted, with collaterals filling the posterior descending (PDA) and posterolateral ventricular (PLV) arteries. Myocardial infarction in the form of hypodensity and areas of calcification were also noted in the RCA territory on CT imaging. The other coronary arteries revealed no significant atherosclerotic disease. A relook at the previous catheter angiography revealed the stent fracture (retrospectively), which was missed during catheter angiography. CTCA on the other hand clearly demonstrated the complex fracture and the current status of the RCA. Aspiration of the pericardial fluid was negative for tuberculosis, and revealed a fibrinoid collection. The patient revealed no viable tissue in the RCA territory on cardiac MRI, and hence further intervention to salvage the RCA supply was deferred. The patient was stable and put on medical therapy and advised regular followups.

## 3. Discussion

Stent fracture is an important and potentially serious complication of drug-eluting stents (DES) [2]. Increasing number of cases with stent fractures are being reported with DES since 2006, especially with 64-slice, or higher, multidetector CT imaging (MDCT). This may be in view of increasing use of DES, greater availability of high-end CT scanners; as well as increasing awareness of this serious complication. Stent fracture (SF) is probably related to mechanical fatigue of the metallic stent strut, which may be aggravated by highly pulsatile structures (myocardial bridge), use of long stents or DES unsupported by neointimal tissue [8]. SF may also result from a manufacturing defect [8]. Various factors that have been implicated for a stent fracture include vessel tortuosity, the presence of a right coronary artery lesion, overlapping stents, and the use of a DES such as a sirolimus-eluting stent [9]. In general stent fractures have been reported to be more common when placed in the right coronary artery (RCA)[1] probably due to its curved course, than in the left anterior descending (LAD) or circumflex (LCX) coronary arteries [1]. The type of stent also influences its risk for fracture. The Cypher stent is more prone to fracture as compared to Taxus and Endeavor stents [1]. In our case, a sirolimus drug eluting stent (ProNova) was used for a significant mid RCA lesion. Longer stents lengths (30 mm) were found to fracture more commonly than shorter (20 mm) stents [10]. Overlapping stents are more likely to fracture rather than isolated stents [1, 9]. There are conflicting reports of association of stent fracture with duration of stent implant. Although positive correlation was found on pathologic analysis by Nakazawa et al. [10], no such association was found in a study by Lim et al. [1]. Other rare complications such as late persistent aneurysms [8] and mycotic aneurysms [11] have also been reported. Medial injury such as that caused by over-distention of the stent has been cited as a cause of in-stent neointimal hyperplasia [12]. Medial injury from aggravated hinge movements at the fracture site and penetration of the sharp edge of a fracture fragment into the lipid core contribute to chronic inflammation which in turn leads to the formation of neointimal hyperplasia [13]. These may play important roles in the adverse effects associated with SF [13–15]. Tenting or early ulceration, seen as a protruding thornlike pouch at the level of the fracture, in patients with mild ISR, supports the theory that medial wall injury arising from the fractured struts is a cause of clinical complications such as ISR, false aneurysm [16], and acute thrombosis.

Nakazawa et al. have graded stent fractures into five categories (Table 1) and concluded that grade V stent fracture was associated with adverse pathologic findings, where as grades I to IV did not have significant impact on the occurrence of adverse pathologic findings such as thrombosis and restenosis [10]. Multidetector CTCA has been found to be more sensitive than conventional coronary angiography in the detection of coronary stent fracture (SF), due to its nearly isotropic multiplanar imaging capabilities, that can depict stents in their long and short axes [1]. MDCT imaging on 64-slice scanners provide most of the relevant details required to assess stents on followup. The important features that must be evaluated in all poststent follow-up cases include; not only the evaluation of in-stent thrombosis, but also features such as stent migration, fracture, buckling, and rarely coronary perforation and aneurysms. Lim et al. conclusively demonstrated that breaks in the rings of the stents, or their interlinks can be demonstrated on 64-row MDCT imaging [1]. When a stent fracture occurs, there is a loss of the normal interspace distance between two consecutive rings, misalignment of the rings as compared to those just above and below it, or complete lack of visualization of the rings. The stent may buckle into the cavity producing small indentations. Stent gaps demonstrated on MDCT probably represent stent fractures in single stents. 46% of stent gaps revealed in-stent stenosis [17]. These are less commonly seen on conventional angiography [17]. Fractures may be partial or complete in nature [1, 8]. According to a study by Lim et al., 33% of stent fractures were missed on conventional angiography [1]. Poor visibility of the struts at fluoroscopy, the lack of familiarity with the appearance of fractures, a lower incidence of SF in these types of stents; have been implicated as reasons for missing stent fractures on conventional angiography. Lim et al. reported missed fractures in all four Taxus devices, during initial conventional coronary angiogram interpretations; all of which were demonstrated on MDCT imaging [1]. Our CT findings showed a loss of strut alignment and a loss of the normal curvature of the RCA stent, along with a localised non-enhancing hypodense peristent collection, suggestive of thrombosis. In our case, secondary thrombosis due to both vascular rupture as well as stent fracture led to complete occlusion of the in-stent RCA, with faint opacification of the acute marginal branch and a short segment of the distal poststent RCA. Collateral circulation was demonstrated with small collaterals from the acute marginal filling the PDA and PLV. Further intervention was deferred as no viable myocardium was identified on delayed enhancement cardiac MRI. Regular followup was advised and the patient was put on medical management for his coronary artery disease, along with antituberculous treatment for a TB knee joint effusion. RCA stent fracture perforating the native vessel with resultant hemopericardium and tamponade has been reported by Choi et al. where emergency coronary bypass grafting was undertaken [18]. Our case also reveals RCA perforation following stent fracture, but did not present as an emergency and the patient continues to do well, in spite of this serious complication.

## 4. Conclusion

64-slice CT coronary angiography is an extremely fast and accurate imaging modality available for the evaluation of stents and their complications. It is found to be more sensitive in detecting stent fractures than conventional catheter angiography. Besides demonstrating in-stent thrombosis, it can effectively evaluate stent fractures, migrations, buckling, or collapse; rare complications such as perforations and aneurysms. This is a rare case of long standing stent fracture with RCA rupture and associated in-stent total occlusion and highlights a unique complication of stent placement: stent fracture causing coronary arterial rupture and subsequent stent migration. It also highlights the utility of 64-row MDCT imaging in effectively demonstrating such serious complications; which may or may not be convincingly shown on conventional catheter angiography.

## Figures and Tables

**Table 1:** 

Grade	Pathology
I	Single strut fracture
II	>2 strut fractures
III	>2 strut fractures with deformation
IV	Transection without gap
V	Transaction causing gap in stent segment.

## References

[1] Lim HB, Hur G, Kim SY (2008). Coronary stent fracture: detection with 64-section multidetector CT angiography in patients and in vitro. *Radiology*.

[2] Makaryus AN, Lefkowitz L, Lee ADK (2007). Coronary artery stent fracture. *International Journal of Cardiovascular Imaging*.

[3] Aoki J, Nakazawa G, Tanabe K (2007). Incidence and clinical impact of coronary stent fracture after sirolimus-eluting stent implantation. *Catheterization and Cardiovascular Interventions*.

[4] Lee MS, Jurewitz D, Aragon J, Forrester J, Makkar RR, Kar S (2007). Stent fracture associated with drug-eluting stents: clinical characteristics and implications. *Catheterization and Cardiovascular Interventions*.

[5] Lee SH, Park JS, Shin DG (2007). Frequency of stent fracture as a cause of coronary restenosis after sirolimus-eluting stent implantation. *American Journal of Cardiology*.

[6] Ehara M, Surmely JF, Kawai M (2006). Diagnostic accuracy of 64-slice computed tomography for detecting angiographically significant coronary artery stenosis in an unselected consecutive patient population: comparison with conventional invasive angiography. *Circulation Journal*.

[7] Nikolaou K, Knez A, Rist C (2006). Accuracy of 64-MDCT in the diagnosis of ischemic heart disease. *American Journal of Roentgenology*.

[8] Vaknin-Assa H, Assali A, Fuchs S, Kornowski R (2007). An unusual cluster of complications following drug-eluted stenting: stent fracture, peri-stent aneurysm and late thrombosis. *Israel Medical Association Journal*.

[9] Choe H, Hur G, Doh JH (2009). A case of very late stent thrombosis facilitated by drug eluting stent fracture: comparative images before and after stent fracture detected by 64-multidetector computed tomography. *International Journal of Cardiology*.

[10] Nakazawa G, Finn AV, Vorpahl M (2009). Incidence and predictors of drug-eluting stent fracture in human coronary artery. A pathologic analysis. *Journal of the American College of Cardiology*.

[11] Singh H, Singh C, Aggarwal N, Dugal JS, Kumar A, Luthra M (2005). Mycotic aneurysm of left anterior descending artery after sirolimus-eluting stent implantation: a case report. *Catheterization and Cardiovascular Interventions*.

[12] Trehan V, Nigam A, Bhamri N (2007). Stent fracture: a biomaterial scientist’s responsibility or an interventionist’s onus?. *Indian Heart Journal*.

[13] Yagi S, Kimura T, Hayashi I, Nishiuchi T (2008). Acute coronary syndrome due to hinge movement of a bare-metal stent. *International Journal of Cardiology*.

[14] Farb A, Weber DK, Kolodgie FD, Burke AP, Virmani R (2002). Morphological predictors of restenosis after coronary stenting in humans. *Circulation*.

[15] Lemos PA, Saia F, Ligthart JMR (2003). Coronary restenosis after sirolimus-eluting stent implantation: morphological description and mechanistic analysis from a consecutive series of cases. *Circulation*.

[16] Harish A, Ezhilan J, Pandurangi UM, Latchumanadhas K, Mullasari Ajit S (2007). Delayed stent fracture and aneurysm formation after sirolimus eluting stent. *Indian Heart Journal*.

[17] Hecht HS, Polena S, Jelnin V (2009). Stent gap by 64-detector computed tomographic angiography. Relationship to in-stent restenosis, fracture, and overlap failure. *Journal of the American College of Cardiology*.

[18] Choi JH, Song B, Song BG (2008). Catastrophic coronary stent fracture and coronary perforation presenting as cardiogenic shock: a rare but fatal late complication of stenting. *Circulation: Cardiovascular Imaging*.

